# Announcement: 4th International Symposium on Applied Bioinorganic Chemistry

**DOI:** 10.1155/MBD.1996.261

**Published:** 1996


### 4th INTERNATIONAL SYMPOSIUM ON APPLIED BIOINORGANIC CHEMISTRY

The Forum, V & A Waterfront, Cape Town, South Africa
**1 - 4 April 1997**

**Introduction**
The 4th International Symposium on Applied Bioinorganic Chemistry will be held in Cape Town, South Africa. This city with its spectacular harbours and majestic Table Mountain is regarded as the gateway to Africa. The newly constructed Waterfront development is a popular destination for both tourists and locals. The wine producing regions of Stellenhosch, Franschhoek and Paarl some 40 km away together with the scenic beaches of the Atlantic and Indian oceans presents an opportunity to experience the Cape hospitality. The symposium will provide a forum for presenting current knowledge on themes such as

- Metal-containing drugs
- Toxicology
- Metalloproteins and enzymes
- Radiopharmaceuticals
- Biologically active chelates and macrocycles
- Biominerals and new materials
- Biotechnology and environmental applications

**Conference venue**
The conference will he held at The Forum in the heart of the popular Victoria & Alfred Waterfront, and is accessible by foot or bus from the "official" conference hotels. There is ample free and paid parking available. The venue allows for a maximum of only 140 participants. Complete, fully paid early registration is therefore essential.

**Speakers**
The following scientists have agreed to present plenary lectures:

| | | | |
|---|---|---|---|
| Prof Kui Wang | CHINA | Prof Jacques Buffle | SWITZERLAND |
| Prof Peter Sadler | UK | Prof Paul Saltman | USA |
| Prof David Williams | UK | Prof Eiichi Kimura | JAPAN |
| Prof Ivano Bertini | ITALY | Prof CC Mjojo | SOUTH AFRICA |

**Conference Fee**
The conference registration fee is R1000.00 for African including South African delegates and US$300 for all other countries. This includes attendance at all scientific sessions, teas, lunches, the Welcoming Reception, "Fruits of the Cape" Buffet, conference dinner, conference programme and book of abstracts. The full amount and your completed registration form should be sent to the Conference Secretariat at the address provided below. Day rates will be provided on request.
The registration fee for delegates who register after 15 January 1997 will be R1200.00 or US$350.
A penalty of R150.00 will be charged, to cover administrative costs, for cancellations received before 15 January 1997. Thereafter, registration fees will not be refunded.
Students and delegates from developing countries may apply for a reduced registration fee. Applications, supported by a curriculum vitae, should be sent to The Treasurer, Dr. Susan Bourne, 4ISABC, at the address provided, by no later than 1 November 1996.

**Accompanying Guests**
The accompanying person registration fee of R500/US$ 110 includes admission to the Welcoming Reception, "Fruits of the Cape" buffet, conference dinner, poster sessions and lunches.

**Presentations**
Type your abstract of no more than 250 words, on an A4 sheet of paper within an area of 240mm x 160mm. Use font size 12pt. **USE BOLD CAPITALS FOR THE TITLE OF YOUR PRESENTATION.** The final book of abstract will be photoreduced to A5 size. Send it to the Conference Secretariat by 15 January 1997. Indicate your preference for a poster or oral presentation. You will be advised by the Scientific Programme Committee whether your abstract has been accepted as an oral or a poster presentation, by no later than 28 February 1997. Oral presentations will be 25 minutes long .

**Registration Desk**
The Conference Secretariat will be available throughout the conference, to assist you with information on the conference programme, airport transport, tours, flight confirmations, late registration, photocopying, telephone and faxing facilities, previewing facilities and general enquiries.

**Language**
The official language of the conference is English. No translation facilities will be provided.

**Social Programme**
An exciting programme is being planned, to complement what promises to be a stimulating symposium. Kindly confirm your participation on the registration form.
A Welcoming Reception will be held on Monday 31 March, at 7pm in the Forum at the V&A.
On Tuesday evening, a "Fruits of the Cape" buffet dinner will be held at the UCT club, on the campus of the University of Cape Town. Enjoy a selection of traditional and modern Cape cuisine, while admiring a panoramic view of the Hottentots Holland mountains. The conference will close with a dinner on Friday 4 April.

**Optional functions and excursions**
No sessions have been scheduled for Wednesday afternoon, to allow delegates to explore the Cape. The following tours have been provisionally booked: • Cape Peninsula and Cape Point and
• Wine Route

**Climate**
At this time of the year, the Western Cape experiences average temperatures of 15-22 °C. Light clothing should be adequate for most of the day and warm sweaters for the evening.

**Transport**
Transport will be provided between the airport and the "official" conference hotels.

**Accommodation**
Rooms have been provisionally reserved at preferential rates, at the following hotels: CENTURION; RITZ INN; CITY LODGE; BREAKWATER LODGE; PORTSWOOD HOTEL.
Final bookings should be negotiated directly between individual **delegates and** the hotel(s) of their choice. **Please quote the** appropriate reference number or the name of the conference (4ISABC) to qualify for the group rate. All contact information for these hotels is provided upon request from the Conference Secretariat.

**Local Organising Committee**

| | | | |
|---|---|---|---|
| H. Andrews | S. Bourne | A. Crouch | T. Demana I. |
| GreenG.E. Jackson | N. Jarvis | K.R. Koch | P.W. Linder |
| I.M. Moodie | S. Wilson | | |

### For more information:

***Scientific Programme***

Prof GE Jackson
University of Cape Town, Private Bag, Rondebosch 7700,
Cape Town, South Africa
Tel: +27.21.650 2531, Fax: +27.21 650 3788, Email: jackson@psipsy.uct.ac.za

***Address Registration forms, Abstracts, and General Enquiries to:***

Conference Secretariat, 4ISABC
Medical Research Council, Francie Vanzijl Drive, Tygerberg 7505, South Africa
Tel: +27.21.938 0433, Fax: +27.21.938 0395, Email: handrews@eagle.mrc.ac.za

